# Evaluating the implementation of the Pediatric Acute Care Education (PACE) program in northwestern Tanzania: a mixed-methods study guided by normalization process theory

**DOI:** 10.1186/s12913-024-11554-3

**Published:** 2024-09-13

**Authors:** Joseph R. Mwanga, Adolfine Hokororo, Hanston Ndosi, Theopista Masenge, Florence S. Kalabamu, Daniel Tawfik, Rishi P. Mediratta, Boris Rozenfeld, Marc Berg, Zachary H. Smith, Neema Chami, Namala P. Mkopi, Castory Mwanga, Enock Diocles, Ambrose Agweyu, Peter A. Meaney

**Affiliations:** 1 Catholic University of Health and Allied Sciences, Mwanza, Tanzania; 2 Pediatric Association of Tanzania, Dar Es Salaam, Tanzania; 3 Hubert Kairuki Memorial University, Dar es Salaam, Tanzania; 4 Stanford University School of Medicine, Palo Alto, CA USA; 5 Area9 Lyceum, Boston, MA USA; 6 Kaiser Permanente, Oakland, CA USA; 7 Muhimbili National Hospital, Dar es Salaam, Tanzania; 8 London School of Hygiene and Tropical Medicine, London, UK

**Keywords:** Adaptive learning, Feasibility, Acceptability, Normalization Process Theory, Implementation Science, Pediatrics, Tanzania

## Abstract

**Background:**

In low- and middle-income countries (LMICs), such as Tanzania, the competency of healthcare providers critically influences the quality of pediatric care. To address this issue, we introduced Pediatric Acute Care Education (PACE), an adaptive learning program to enhance provider competency in Tanzania’s guidelines for managing seriously ill children. Adaptive learning is a promising alternative to current in-service education, yet optimal implementation strategies in LMIC settings are unknown.

**Objectives:**

(1) To evaluate the initial PACE implementation in Mwanza, Tanzania, using the construct of normalization process theory (NPT); (2) To provide insights into its feasibility, acceptability, and scalability potential.

**Methods:**

Mixed-methods study involving healthcare providers at three facilities. Quantitative data was collected using the Normalization MeAsure Development (NoMAD) questionnaire, while qualitative data was gathered through in-depth interviews (IDIs) and focus groups discussions (FGDs).

**Results:**

Eighty-two healthcare providers completed the NoMAD survey. Additionally, 24 senior providers participated in IDIs, and 79 junior providers participated in FGDs. Coherence and cognitive participation were high, demonstrating that PACE is well understood and resonates with existing healthcare goals. Providers expressed a willingness to integrate PACE into their practices, distinguishing it from existing educational methods. However, challenges related to resources and infrastructure, particularly those affecting collective action, were noted. Early indicators point toward the potential for long-term sustainability of the PACE, but assessment of reflexive monitoring was limited due to the study’s focus on PACE’s initial implementation.

**Conclusion:**

This study offers vital insights into the feasibility and acceptability of implementing PACE in a Tanzanian context. While PACE aligns well with healthcare objectives, addressing resource and infrastructure challenges as well as conducting a longer-term study to assess reflexive monitoring is crucial for its successful implementation. Furthermore, the study underscores the value of the NPT as a framework for guiding implementation processes, with broader implications for implementation science and pediatric acute care in LMICs.

**Supplementary Information:**

The online version contains supplementary material available at 10.1186/s12913-024-11554-3.

## Contributions to the literature

*Introduces PACE*: This study uniquely evaluated the PACE program in a low-resource setting, offering initial evidence on its implementation and potential impact on pediatric care.

*Utilizes the NPT framework*: By employing a NPT framework, this research provides a novel methodological example of how to assess the incorporation of e-learning in LMIC clinical settings.

*Informs Implementation Strategies*: These findings contribute to the design of effective e-learning strategies for healthcare education in LMICs, suggesting practical steps for broader application.

*Expands Local Capacity*: Demonstrates how PACE can build local healthcare capacity, informing ongoing efforts to sustainably improve pediatric care through education in similar environments.

## Background

### Context and importance of the study

Pediatric in-service education for healthcare providers in Low- and Middle-income countries (LMICs) often lacks reach, effectiveness, and sustainability, contributing to millions of child deaths annually [1, 2]. Pneumonia, birth asphyxia, dehydration, malaria, malnutrition, and anemia cause over 4 million child deaths annually, with half occurring in sub-Saharan Africa and thousands in Tanzania [3, 4]. The Tanzanian government aims to reduce neonatal mortality from 20/100,000 to the Sustainable Development Goals (SDGs) target of 12/100,000 by 2030 [5].

### Brief review of the literature

Provider knowledge and skills competency are crucial for care quality in LMICs [2, 6]. However, conventional in-service education methods are often inadequate and unsustainable [6]. These methods do not adapt to individual providers’ knowledge or schedules, target minimal competency, and lack long-term refresher learning, limiting their effectiveness [7–10].

Adaptive learning can address these limitations by customizing the timing and sequence of combined e-learning and in-person skills training, creating individualized pathways that reinforce learning and enhance skills competency. This approach helps mitigate manpower and resource shortages in LMICs and represents a strategic innovation in knowledge dissemination.

The World Health Organization (WHO) emphasizes the importance of e-learning solutions for healthcare workers globally [11]. Adaptive learning, with its capacity to adjust to individual needs, holds significant promise for enhancing training efficiency. However, formal studies on adaptive learning in LMIC contexts are scarce. Establishing best practices in e-learning and adaptive methodologies will enhance the dissemination of evidence-based interventions and improve clinical practice and patient outcomes.

To address current educational limitations for healthcare workers in LMICs, we developed the Pediatric Acute Care Education (PACE) program [12, 13]. This adaptive e-learning program offers 340 learning objectives across 10 assignments, covering newborn and pediatric care guidelines for management of seriously ill children. The PACE program’s implementation strategy includes an adaptive e-learning platform optimized for mobile phones, a steering committee, a full-time PACE coordinator, and an escalating nudge strategy to encourage participation.

### Study aims and objectives

The primary aim of this research is to assess the preliminary implementation of the PACE intervention across two types of pediatric acute care facilities: zonal hospitals and health centers. The study has two principal objectives: (1) To evaluate the initial PACE implementation in Mwanza, Tanzania, using the constructs of Normalization Process Theory (NPT); (2) To provide insights into its feasibility, acceptability, and scalability potential.

## Methods

### Study design

This study employed a mixed methods approach to evaluate the implementation of the PACE program in three healthcare settings in northwestern Tanzania, nested within a larger pilot implementation of PACE within eight health facilities of the Pediatric Association of Tanzania’s Clinical Learning Network. The study utilized NPT as a framework, combining quantitative and qualitative methods. Quantitatively, a tailored NoMAD survey instrument evaluates the integration of PACE into routine clinical practice. Qualitatively, in-depth interviews and focus group discussions enrich the data.

### Theoretical framework

**Fig. 1:**
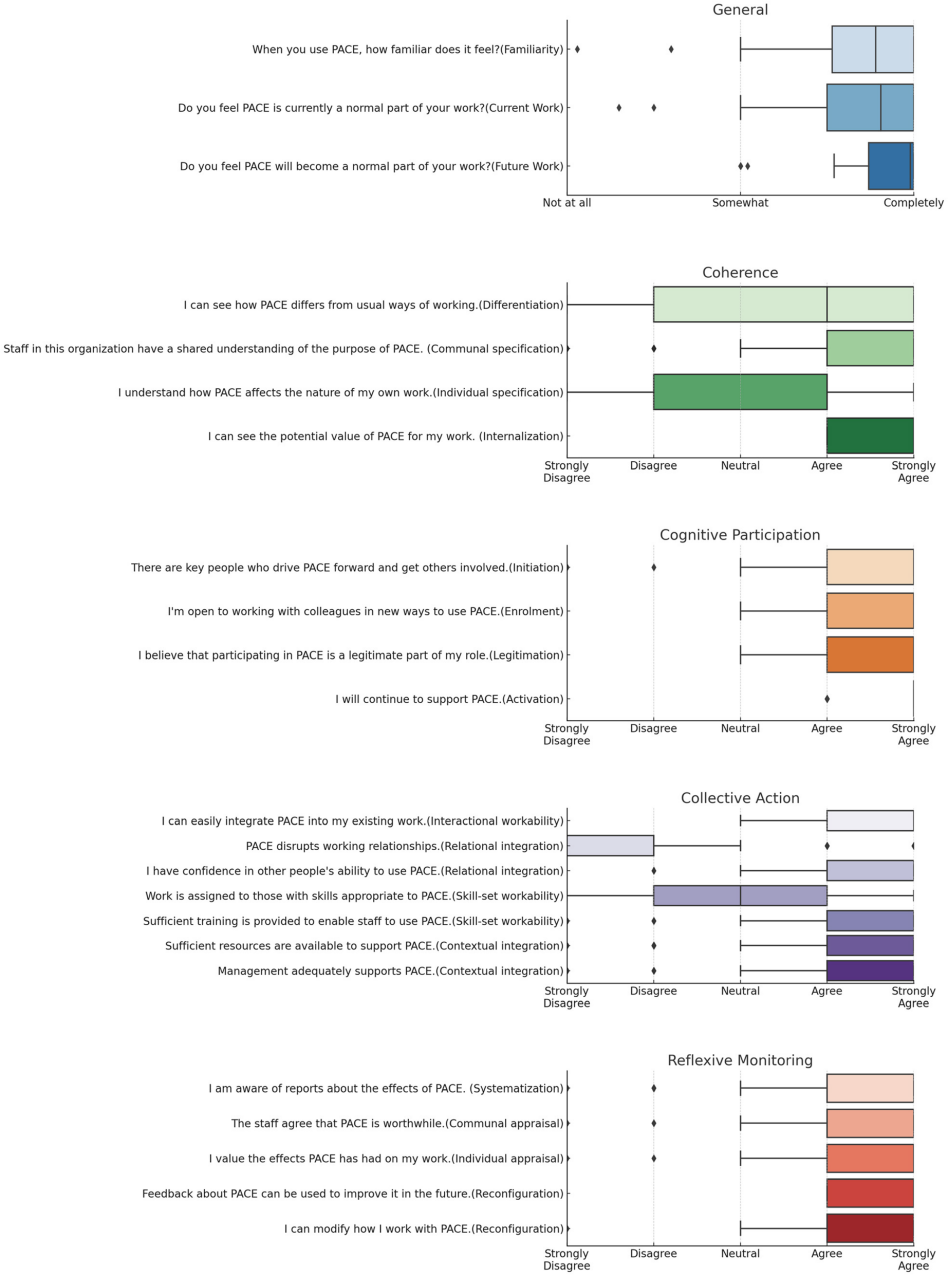
Boxplot of participant responses to NoMAD survey by NPT construct and subconstruct

NPT has been described as a sociological toolkit for helping us understand the dynamics of implementing, embedding, and integrating new technology or a complex intervention into routine practice [14]. NPT provides a conceptual framework for understanding and evaluating the processes (implementation) by which new health technologies and other complex interventions are routinely operationalized in everyday work (embedding) and sustained in practice (integration) [15–20]. The theory is organized around four main constructs, each of which has its own subconstructs [15]. These constructs collectively offer insights into the feasibility, acceptability, and scalability of an intervention or innovation (Fig. 1). Each of these constructs and subconstructs offers a unique lens through which the feasibility, acceptability, and scalability of a new practice can be evaluated, thereby aiding in its effective implementation.

### Study setting

The study was conducted between August 2022 and July 2023 at three healthcare facilities in Mwanza, Tanzania. The Bugando Medical Centre (BMC), an urban zonal referral and teaching hospital, sees about 7,000 births per year and 6,550 pediatric admissions per year for children aged 1 month to 5 years; the urban Makongoro Health Centre, handles approximately 359 births per year but refers newborn and pediatric admissions to the nearby regional or zonal hospital; and the rural Igoma Health Centre sees about 3,850 births per year and 959 pediatric admissions per year for children aged 1 month to 5 years.

### Providers

#### Eligibility criteria

Providers included in the study were required to have a minimum command of English and be actively providing pediatric care to sick patients at least part-time. Eligible providers encompassed a wide range of professional cadres, reflecting the diversity of healthcare providers in Tanzania. These included specialists (medical officers with 3 additional years of specialization), medical officers (5 years of education and 1-year internship), nursing officers (4 years of education and 1-year internship), assistant medical officers (clinical officers with 2 additional years of clinical training), assistant nursing officers (3 years of education), clinical officers (3 years of education), clinical assistants (2 years of education), enrolled nurses (2 years of education), and medical attendants (1 year of education). In addition to providers, senior facility staff with administrative roles who supervise PACE providers, such as ward matrons, medical officers-in-charge, and nursing officers-in-charge, were eligible to participate. The bulk of the care is provided by junior medical officers and nurses, who have limited training and experience caring for children with severe illnesses

#### Recruitment process

Healthcare providers were informed about the study through their facility leaders, and individuals who responded to the survey were not necessarily the same as those who participated in the focus groups or in-depth interviews.

### Data collection tools

#### NoMAD questionnaire

The NoMAD is a 23-item questionnaire based on the NPT that was designed to assess the social processes influencing the integration of complex interventions [18, 21]. It includes 3 general items and 20 related to specific NPT constructs (4 Coherence, 7 Collective Action, 4 Cognitive Participation, 5 Reflexive Monitoring). The general items were scored on a scale of 0-100, and the NPT construct items were modified to include a five-point Likert scale (1-Strongly Agree, 5-Strongly Disagree) and additional options for respondents to indicate whether a question was not relevant to their role, stage, or intervention itself. The NPT subconstruct survey items are listed in Table 1, and the complete survey is provided in the Supplementary Materials.

**Table 1:** Normalization process theory constructs and subconstructs

**NPT construct** [20]	**Sub-construct** [15]	**Description** [22]
**Coherence:** How do people work together to understand and plan the activities that need to be accomplished to put an intervention and its components into practice?	**Differentiation**	**Description:** How do people distinguish interventions and their components from their current ways of working?
	**Communal specification**	**Description:** How do people collectively agree about the purpose of interventions and their components?
	**Individual specification**	**Description:** How do people individually understand what interventions and their components require of them?
	**Internalization**	**Description:** How do people construct potential value of interventions and their components for their work?
**Cognitive participation:** How do people work together to create networks of participation and communities of practice around interventions and their components?	**Initiation**	**Description:** How do key individuals drive interventions and their components forward?
	**Enrolment**	**Description:** How do people join in with interventions and their components?
	**Legitimation**	**Description:** How do people agree that interventions and their components are the right thing to do and should be part of their work?
	**Activation**	**Description:** How do people continue to support interventions and their components?
**Collective action:** How do people work together to enact interventions and their components?	**Interactional workability**	**Description:** How do people do the work required by interventions and their components?
	**Relational integration**	**Description:** How does using interventions and their components affect the confidence that people have in each other?
	**Skill-set workability**	**Description:** How is the work of interventions and their components appropriately allocated to people?
	**Contextual integration**	**Description:** How is the work of interventions and their components supported by host organizations?
**Reflexive monitoring** **Description:** How do people work together to appraise interventions and their components?	**Systematization**	**Description:** How do people access information about the effects of interventions and their components?
	**Communal appraisal**	**Description:** How do people collectively assess interventions and their components as worthwhile?
	**Individual appraisal**	**Description:** How do people individually assess interventions and their components as worthwhile?
	**Reconfiguration**	**Description:** How do people modify their work in response to their appraisal of interventions and their components?

#### In-depth interviews (IDIs) and focus group discussions (FGDs)

Interview guides were developed based on previous experience with similar data collection tools. The training and pretesting of the tools were conducted by the study investigators.

### Data collection process

#### NoMAD survey

All PACE participants were invited via WhatsApp to complete the NoMAD survey directly in REDCap, 30 days post-intervention or upon completion of the PACE course.

#### Focus group discussions and in-depth interviews

We employed a purposeful sampling strategy for the qualitative components, selecting senior healthcare providers for in-depth interviews (IDIs) and junior providers for focus group discussions (FGDs). This approach ensured junior providers felt comfortable speaking openly, avoiding inhibition from senior participants in focus groups, and facilitated methodological triangulation to enhance the credibility and validity of the findings. Data was triangulated using three different types: methodological triangulation with IDIs and FGDs, investigator triangulation with different research assistants collecting data, and data triangulation using data from IDIs, FGDs and NoMAD surveys. Data collection began with a series of field visits, guided by NPT constructs, and included IDIs and FGDs. FGDs, segregated by sex but including a mix of cadres from each health facility, enriched the diversity of perspectives. The iterative nature of our methodology allowed for continuous refinement of our theoretical framework, methodologies, and sampling strategies, informed by emerging data. Consequently, the guides for both the IDIs and FGDs were dynamically modified to reflect the evolving study themes. All sessions, including IDIs and FGDs, were conducted in Kiswahili at the providers’ work premises, adding contextual depth. The IDI and FGD interview guides were originally developed in English, translated into Kiswahili (the national language), and then back translated into English to ensure that the meaning was retained. Both IDIs and FGDs were meticulously audio-recorded, transcribed verbatim, and then translated into English for analysis. Back-translation was employed to ensure validity.

### Data analysis

#### Quantitative analysis

Descriptive statistics are reported as frequencies and percentages or medians and interquartile ranges, with comparisons via Fisher’s exact test or the Mann‒Whitney U test as appropriate. Analyses were conducted using Stata 17.0 (Stata Corp, College Station, TX, USA).

#### Qualitative analysis

The analysis process, conducted concurrently with data collection, was instrumental in achieving theoretical saturation, marked by the cessation of new information from ongoing IDIs and FGDs. To ensure the validity and depth of our findings, we implemented member checking and investigator triangulation, with two independent investigators coding and interpreting the data using NVivo 2020 software (QSR International Pty Ltd., Sydney, Australia). This software facilitated a hybrid coding approach in which blended deductive and inductive methods were used for comprehensive thematic content analysis. Contextual insights from the IDIs and FGDs were key to interpreting the findings, with representative quotations included to illustrate the identified themes. Data triangulation was achieved using diverse data sources, and the research team’s expertise further enhanced the rigor and reflexivity of the analysis.

#### Summary of feasibility, acceptability and scalability

We used the Proctors definition of implementation outcomes and mapped the NoMAD survey results to NPT subconstructs using the definition of May et al. [22, 23].

*Feasibility* is concerned with the practical aspects of implementing a new intervention, including resource allocation, training, and ease of integration into existing work. In the NPT, this aligns closely with the construct of “collective action,” which refers to the operational work that people do to enact a set of practices. To assess feasibility, we interpreted our responses as follows: “Sufficient training is provided to enable staff to use PACE” (collective action, skill set workability); “Sufficient resources are available to support PACE”; “Management adequately supports PACE” (collective action, contextual integration); and “I can easily integrate PACE into my existing work” (collective action, interactional workability).

*Acceptability* refers to the extent to which the new intervention is agreeable or satisfactory among its users. To assess acceptability, we interpreted our responses as follows: “Staff in this organization have a shared understanding of the purpose of PACE” (coherence: communal specification); “I believe that participating in PACE is a legitimate part of my role” (cognitive participation, legitimation); “The staff agree that PACE is worthwhile” (reflexive monitoring, communal appraisal); and “I value the effects PACE has had on my work” (reflexive monitoring, individual appraisal). In addition, we compared scores between zonal hospitals and health centers.

*Scalability* involves the ability to expand the intervention to other settings while maintaining its effectiveness. To assess scalability, we interpreted our responses as “I will continue to support PACE” (cognitive participation, activation); “Work is assigned to those with skills appropriate for PACE” (collective action, skill set workability); “feedback about PACE can be used to improve it in the future”; and “I can modify how I work with PACE” (reflexive monitoring, reconfiguration).

#### Ethical considerations

All the providers provided informed consent, and the study was approved by the Institutional Review Board of the Tanzania National Institute of Medical Research (NIMR/HO/R.8a/Vol. IX/3990), Stanford University (60379), the ethics committee of the Catholic University of Health and Allied Science (no ID number given), and the Mwanza Regional Medical Officer (Ref. No. AG.52/290/01A/115).

#### Techniques to enhance trustworthiness

Techniques to enhance trustworthiness included a purposeful sampling strategy, meticulous data collection in Kiswahili with back-translation, and the use of methodological, investigator, and data triangulation [24]. The analysis process was iterative and concurrent with data collection, employing hybrid coding and member checking to ensure systematic, explicit, and reproducible findings.

#### Reporting guidelines

This study adheres to the STROBE and SRQR reporting guidelines for comprehensive and explicit reporting of observational and qualitative studies, respectively [25, 26].

## Results

### Provider demographics

Eighty-two of the 272 eligible healthcare providers from the three facilities completed the NoMAD survey, resulting in a 30% response rate. Of the 82 respondents, 59 were from zonal hospitals and 23 from health centers (Table 2). The median ages were 27 and 29 years for zonal hospital and health center staff, respectively. The gender distribution was similar in both settings, with 39% female in the zonal hospital group and 43.5% in the health centers.

**Table 2:** NoMAD respondent characteristics

**Characteristics**	**Overall**	**Zonal Hospital**	**Health Center**	***p* value**
n	82	59	23	
Age (years) median [IQR]	27.0 [6.75]	27.0 [3.0]	29.0 [8.0]	N/S
Female, n (%)	33 (40.2%)	23 (39.0%)	10 (43.5%)	N/S
Cadre, n (%)				< 0.05
Nursing officer	32 (39.0%)	25 (42.4%)	7 (30.4%)	
Medical officer	30 (36.6%)	28 (47.5%)	2 (8.7%)	
Other	8 (9.8%)	4 (6.8%)	4 (17.4%)	
Clinical officer	7 (8.5%)		7 (30.4%)	
Physician (specialist/superspecialist)	2 (2.4%)	2 (3.4%)		
Medical attendant	2 (2.4%)		2 (8.7%)	
Assistant medical officer	1 (1.2%)		1 (4.3%)	
Clinical experience (years), median [IQR]	2.0 [4.0]	1.0 [4.0]	4.0 [9.0]	<0.05
Any previous newborn or pediatric in-service education, n (%)	59.0 (72.0%)	42.0 (71.2%)	17.0 (73.9%)	N/S
Overall job satisfaction (1-5), mean (std)	3.7 (0.9)	3.8 (0.8)	3.4 (1.0)	N/S

There were significant differences in cadre distribution: zonal hospitals had more medical staff (47.5% vs. 8.7%) and nurses (42.4% vs. 30.4%), while health centers had more clinical officers (30.4% vs. 0%). Clinical experience also varied, with a median of 1 year at zonal hospitals and 4 years at health centers (*p =* 0.004). Previous participation in newborn or pediatric in-service education (e.g., Helping Babies Breathe, Helping Children Survive) was similar across the facilities, ranging from 71% to 73%. Job satisfaction scores did not significantly differ between the two groups.

A total of seventy-nine healthcare providers participated in IDIs or FGDs. Twenty-four senior providers completed IDIs, 18 from the zonal hospital and 6 from health centers., 13 FGDs with an average of 4 junior providers per group were conducted to achieve thematic saturation, including 39 participants from zonal hospitals and 16 from health centers. The represented cadres included medical officers (26, 32.9%), nurses (19, 24.1%), interns (16, 20.3%), clinical officers (12, 15.2%), assistant medical officers (3, 3.8%), and medical attendants (3, 3.8%). Clinical experience among participants ranged from 1 to 20 years. Compared to the NoMAD survey, participants in IDIs and FGDs included a higher proportion of medical officers (including interns) and clinical officers, but a lower proportion of nursing officers and other cadres.

### NoMAD survey results

#### General items

Familiarity and general satisfaction with PACE were high, with median scores of 89 and 91, respectively, and both showed moderate, balanced variability (interquartile ranges of 76-100 and 75-100, respectively) (Table 3, Fig. 1). Optimism for the future use of PACE was highest, with a median score of 99 and narrow variability (87-100), indicating a strong skew towards higher scores. No significant differences were observed between the zonal hospitals and health centers.

**Table 3:** NoMAD survey data table

**NPT construct** [20]	**Sub-construct** [15]	**NOMAD survey item**	**Descriptive statistics**						**Comparative statistics**				
			**count**	**min**	**25%**	**50%**	**75%**	**max**	**Zonal Hospital**		**Health center**		***p*-values**
									**Median**	**[IQR]**	**Median**	**[IQR]**	
**General**	**Familiarity**	When you use PACE, how familiar does it feel?	74	3	76	89	100	100	90	76-100	89	82-100	0.49
	**Current Work**	Do you feel PACE is currently a normal part of your work?	74	15	75	90.5	100	100	90.5	72-100	91.5	75-100	0.76
	**Future Work**	Do you feel PACE will become a normal part of your work?	73	50	87	99	100	100	99	87-100	99	87-100	0.66
**Coherence:** How do people work together to understand and plan the activities that need to be accomplished to put an intervention and its components into practice?	**Differentiation**	I can see how PACE differs from usual ways of working.	80	1	1	2	4	5	2	1-4	2	1-4	0.97
	**Communal specification**	Staff in this organization have a shared understanding of the purpose of PACE.	79	1	1	2	2	5	2	2-2	2	1-2	0.06
	**Individual specification**	I understand how PACE affects the nature of my own work.	81	1	2	2	4	5	2	2-4	2	1-4	0.78
	**Internalization**	I can see the potential value of PACE for my work.	79	1	1	1	2	2	1	1-2	1	1-2	0.92
**Cognitive participation:** How do people work together to create networks of participation and communities of practice around interventions and their components?	**Initiation**	There are key people who drive PACE forward and get others involved.	78	1	1	1	2	5	1	1-2	2	1-2	0.19
	**Enrolment**	I’m open to working with colleagues in new ways to use PACE.	79	1	1	1	2	3	1	1-2	1	1-2	0.53
	**Legitimation**	I believe that participating in PACE is a legitimate part of my role.	81	1	1	1	2	3	1	1-2	1	1-2	0.67
	**Activation**	I will continue to support PACE.	80	1	1	1	1	2	1	1-2	1	1-1	0.25
**Collective action:** How do people work together to enact interventions and their components?	**Interactional workability**	I can easily integrate PACE into my existing work.	81	1	1	1	2	3	1	1-2	1	1-2	0.30
	**Relational integration**	PACE disrupts working relationships.	74	1	4	4	5	5	4	4-5	4	3-5	0.02
		I have confidence in other people’s ability to use PACE.	78	1	1	2	2	4	2	1-2	1	1-2	0.15
	**Skill-set workability**	Work is assigned to those with skills appropriate to PACE.	73	1	2	3	4	5	3	2-4	2.5	1.5-4	0.93
		Sufficient training is provided to enable staff to use PACE.	81	1	1	2	2	5	2	1-2	2	1-2	0.48
	**Contextual integration**	Sufficient resources are available to support PACE.	79	1	1	2	2	5	2	1-2	1	1-2	0.24
		Management adequately supports PACE.	79	1	1	2	2	5	2	1-2	2	1-2	0.77
**Reflexive monitoring** **Description:** How do people work together to appraise interventions and their components?	**Systematization**	I am aware of reports about the effects of PACE.	77	1	1	2	2	5	2	1-2	2	1-2	0.72
	**Communal appraisal**	The staff agree that PACE is worthwhile.	80	1	1	2	2	5	2	1-2	2	1-2	0.36
	**Individual appraisal**	I value the effects PACE has had on my work.	79	1	1	1	2	5	1	1-2	2	1-2	0.13
	**Reconfiguration**	Feedback about PACE can be used to improve it in the future.	81	1	1	1	2	2	1	1-2	1	1-2	0.29
		I can modify how I work with PACE.	81	1	1	2	2	5	1	1-2	2	1-2	0.45

#### NPT constructs

##### Coherence

Providers reported understanding how to work together and plan the activities to put PACE and its components into practice. Strong agreement on the value of PACE is indicated by the median score for “Internalization" (1, “strongly agree,” IQR [1, 2]) (Table 3, Fig. 1). Agreement on PACE’s purpose and its differentiation from existing work is indicated by the median scores for “Communal Specification” (2, “agree,” IQR [1, 2]), “Differentiation” (2, “agree,” IQR [1, 4]) and “Individual Specification” (2, “agree,” IQR [2, 4]), respectively. No significant differences were observed between the zonal hospitals and health centers.

##### Cognitive participation

Providers reported understanding how to work together to create networks of participation and communities of practice around PACE and its components. Strong agreement for ongoing PACE support, PACE participation and leadership, and PACE integration into work is indicated by the median scores for “Activation” (1, “strongly agree,” IQR [1, 1]), “Enrollment” (1, “strongly agree,” IQR [1, 1]), “Initiation” (1, “strongly agree,” IQR [1, 1]), and “Legitimation” (1, “strongly agree,” IQR [1, 2]), respectively. Narrow IQRs highlight the homogeneous support among providers. No significant differences were observed between the zonal hospitals and health centers.

##### Collective action

Providers reported understanding how to work together to enact PACE and its components, with greater certainty of not disrupting working relationships in the zonal hospital compared to health centers. Strong agreement that the work required by PACE is manageable, has sufficient training and resources, and receives strong organizational support is indicated by the median scores for “Interactional Workability” (1, “strongly agree,” IQR [1, 1]), “Skill-set Workability” (1, “strongly agree,” IQR [1, 2]) and “Contextual Integration” (2, “agree,” IQR [2, 2]) (Table 3, Fig. 1). Agreement that PACE does not disrupt working relationships is indicated by the median score for “Relational Integration” (4, “disagree,” IQR [4, 5]). Zonal hospital providers had significantly less variability that PACE would not disrupt working relationships (relational integration)compared to health centers(IQR [4, 5] vs [3, 5] *p=*0.02).

##### Reflexive monitoring

Providers reported understanding how to work together to evaluate the benefits of PACE and its components. Strong agreement on how people individually assess the value of PACE is indicated by the median score for “Individual Appraisal” (1, “strongly agree,” IQR [1, 1]). Agreement on how people access information to assess the value of PACE, how to value PACE collectively, and work adjustments needed for PACE is indicated by the median scores for “Systematization” (2, “agree,” IQR [2, 2]), “Communication Appraisal” (2, “agree,” IQR [2, 2]), and “Reconfiguration” (2, “agree,” IQR [2, 2]) (Table 3, Fig. 1). No significant differences were observed between the zonal hospitals and health centers.

### IDI and focus group results

#### Coherence themes

Providers value PACE for its detailed guidance on specific pediatric cases, such as difficulty breathing, which was not covered in their basic training (Table 4). PACE is seen as a tool for empowering providers to reduce child mortality and improve service quality, aligning with facility goals. Providers believe that PACE has enhanced their understanding and management of seriously ill children. They find that PACE is consistent with Tanzanian and WHO guidelines and useful both in their work and in training medical students.

**Table 4:** Thematic summary of key perspectives from focus group discussions and in-depth interviews

**NPT construct**^1^	**Sub-construct**^2^	**Description**^3^
**Coherence:** How do people work together to understand and plan the activities that need to be accomplished to put an intervention and its components into practice?	**Differentiation**	**Description:** How do people distinguish interventions and their components from their current ways of working? Providers value PACE for its detailed guidance on specific pediatric cases, such as difficulty breathing, which was not covered in their basic training. One provider said, "PACE goes beyond basic knowledge, offering detailed steps for managing cases like difficulty breathing. This is a significant advantage."
	**Communal specification**	**Description:** How do people collectively agree about the purpose of interventions and their components? PACE is seen as a tool for empowering providers to reduce child mortality and improve service quality, aligning with facility goals. One provider remarked, “PACE aims to empower us to reduce child mortality,” while another noted, “Its objectives align with our hospital’s goals to update healthcare providers’ knowledge.”
	**Individual specification**	**Description:** How do people individually understand what interventions and their components require of them? Providers believe PACE has enhanced their understanding and management of seriously ill children. One provider observed, "Before PACE, I relied on existing procedures and guidelines. Now, I’ve gained new insights that could positively impact our treatment system."
	**Internalization**	**Description:** How do people construct potential value of interventions and their components for their work? Providers find that PACE is consistent with Tanzanian and WHO guidelines, useful both in their work and in training medical students. One provider said, "PACE refreshes my memory and aligns with existing guidelines. I use it to educate medical students and junior doctors."
**Cognitive participation:** How do people work together to create networks of participation and communities of practice around interventions and their components?	**Initiation**	**Description:** How do key individuals drive interventions and their components forward? Providers were introduced to PACE by colleagues and supervisors, prompting them to enroll. One provider said, "Specialists introduced us to PACE, and we started learning." Another noted, "After seeing a colleague engage with PACE, I joined too." Some providers find individual initiation beneficial, as one stated, "Starting alone is effective."
	**Enrolment**	**Description:** How do people join in with interventions and their components? Providers mainly use PACE individually but also share modules to spread knowledge. One provider said, "I often use PACE on my phone but also share modules with colleagues."
	**Legitimation**	**Description:** How do people agree that interventions and their components are the right thing to do and should be part of their work? PACE is seen as empowering providers to enhance their pediatric care. One provider noted, "PACE taught me how to administer oxygen based on a child’s age." Another highlighted PACE's flexibility, saying, "You can engage with PACE individually or in groups."
	**Activation**	**Description:** How do people continue to support interventions and their components? Despite busy schedules, providers are committed to PACE training. One provider stated, "Our commitment helps translate knowledge into practice." Another emphasized their personal dedication, saying, "I find time to study PACE multiple times a week, showing my commitment."
**Collective action:** How do people work together to enact interventions and their components?	**Interactional workability**	**Description:** How do people do the work required by interventions and their components? PACE’s digital format allows for individual study and facilitates group discussions. One provider noted, "PACE’s digital nature allows for flexible study schedules." Group discussions often occur in the mornings, as another provider said, "We discuss PACE modules early before attending to patients."
	**Relational integration**	**Description:** How does using interventions and their components affect the confidence that people have in each other? Initially, providers engaged with PACE for personal benefit but later saw the value in sharing knowledge. One provider stated, "I initially used PACE for personal growth but later realized the importance of sharing this knowledge." Teamwork and collective benefits were emphasized, with one provider noting, "We work as a team to meet our objectives."
	**Skill-set workability**	**Description:** How is the work of interventions and their components appropriately allocated to people? Providers value the practical application of PACE knowledge in patient care. One provider said, "After learning, it’s crucial to apply this knowledge in treating patients."
	**Contextual integration**	**Description:** How is the work of interventions and their components supported by host organizations? Challenges like inadequate supplies and lack of electricity hinder PACE implementation. One provider stated, “‘’Sometimes we face difficulties such as inadequate supply of medical equipment and supplies. For example, there is a child in need of oxygen while there is no electricity and we do not have standby generator. This becomes a barrier to translating PACE in practice." However, the availability of tools and support from PACE management facilitates implementation, as another provider noted, "Availability of tools and support has eased PACE's translation into practice.”
**Reflexive monitoring** **Description:** How do people work together to appraise interventions and their components?	**Systematization**	**Description:** How do people access information about the effects of interventions and their components? No quotes directly address this subconstruct, suggesting a need for further exploration within the PACE context.
	**Communal appraisal**	**Description:** How do people collectively assess interventions and their components as worthwhile? Providers find PACE valuable for educating junior doctors, simplifying complex topics, and boosting confidence. One provider noted, "PACE aids in teaching junior doctors by simplifying complex topics and enhancing my confidence during discussions."
	**Individual appraisal**	**Description:** How do people individually assess interventions and their components as worthwhile? Providers believe PACE has enriched their knowledge and confidence in pediatric care. Quotes summarizing this sentiment include, “I've gained confidence and can act quickly in emergencies,” and “I can provide timely service with increased courage.”
	**Reconfiguration**	**Description:** How do people modify their work in response to their appraisal of interventions and their components? A notable challenge is the inaccessibility of learned material for future reference, hindering providers’ ability to refresh their knowledge. One provider stated, "Once you complete a module, it becomes inaccessible, making it difficult to revisit for future case management."

#### Cognitive participation themes

Providers were introduced to PACE by colleagues and supervisors, prompting them to enroll (Table 4). They mainly use PACE individually but also share modules to spread knowledge. PACE is seen as empowering providers to enhance their pediatric care. Despite busy schedules, providers are committed to PACE training.

#### Collective action themes

PACE's digital format allows for individual study and facilitates group discussions (Table 4). Initially, providers engaged with PACE for personal benefit but later saw the value in sharing knowledge. Providers value the practical application of PACE knowledge in patient care. Challenges like inadequate supplies and a lack of electricity hinder PACE implementation, but the availability of tools and support from PACE management facilitates implementation.

#### Reflexive monitoring themes

Providers find PACE valuable for educating junior doctors, simplifying complex topics, and boosting confidence (Table 4). They believe that PACE has enriched their knowledge and confidence in pediatric care. A notable challenge is the inaccessibility of learned material for future reference, hindering providers’ ability to refresh their knowledge.

### Summary of feasibility, acceptability, and scalability

Overall, data from NoMAD survey responses indicated that PACE is generally feasible across healthcare settings, with providers either agreeing or strongly agreeing that people do the work required by interventions and their components (interactional workability median 1 “strongly agree” [1, 2]) or that the work of interventions and their components is supported by host organizations (contextual integration median 2 “agree” [1, 2]).

Furthermore, NoMAD survey responses indicated that PACE is also generally acceptable among healthcare providers. Providers collectively agreed about the purpose of PACE and its components (communal specification median 2 “agree” [1, 2]), agreed that PACE and its components are the right thing to do and should be part of their work (legitimation median 1 “strongly agree” [1, 2]), and collectively and individually agreed that PACE is worthwhile (communal appraisal median 2 “agree” [1, 2]; individual appraisal median 1 “strongly agree” [1, 2]).

Lastly, NoMAD survey responses indicated that PACE appears to be scalable, with some variability in its adaptability and skill-set alignment. Providers strongly agreed that they would continue to support PACE and its components (activation median 1 “strongly agree” [1, 1]), that they could modify their work in response to their appraisal of PACE, and that feedback could be used to improve it in the future (reconfiguration median 1 “strongly agree” [1, 2]). Providers agreed or were neutral about the work of PACE and its components being appropriately allocated to people (skill-set workability median 3 “neutral” [2, 4]), indicating that additional work is needed to identify the correct providers to participate in PACE or that additional support needs to be allocated to those providers to complete PACE.

## Discussion

This mixed-methods pilot study explored the feasibility, acceptability, and scalability of the PACE intervention among healthcare providers in Mwanza, Tanzania, using the NPT framework. The study demonstrated that PACE is generally well understood, aligns with existing healthcare goals, and is feasible to providers. There was strong acceptance and understanding that PACE should become part of normal work. Challenges to scalability lie in ensuring adequate resource and infrastructure support. Qualitative data from IDIs and FGDs enriched the findings by providing detailed insights that supported and contrasted with the NoMAD survey results, highlighting both the strengths and challenges of implementing PACE in a resource-limited setting.

### Interpretation of findings

The study demonstrated that PACE is feasible. It is generally well understood by healthcare providers and aligns with existing healthcare goals. Providers found PACE to be practical in enhancing their ability to manage pediatric cases, particularly those not adequately covered in their basic training. For instance, providers appreciated the detailed guidance PACE offers for managing conditions like difficulty breathing, which they found invaluable. This alignment with healthcare objectives, such as focusing on improving service quality of newborn and child acute care to reduce child mortality underscores PACE’s potential for integration into routine clinical practice. The fact that PACE aligns with both Tanzanian and WHO guidelines further reinforces its relevance and applicability in the local healthcare context.

Provider training programs that focus on improving specific clinical performance objectives tend to yield better outcomes compared to those that cover broad topics. Targeted training programs, such as those designed to enhance specific clinical skills, have been shown to significantly improve the competency and confidence of healthcare providers. For instance, a systematic review we conducted in 2010 demonstrated that provider education programs in LMICs that focused on the needs and resources of the local healthcare environment had greater effectiveness [8]. Similarly, a study by Bluestone et al. (2013) found that focused training in neonatal resuscitation improved the performance of healthcare providers in emergency situations, as evidenced by increased neonatal survival rates [27]. In contrast, broad-topic training programs, while valuable for general knowledge enhancement, often lack the specificity needed to address critical clinical skills gaps effectively. As highlighted by Frenk et al. (2010), broad educational approaches may not adequately prepare providers for the complex, high-stakes situations they encounter in practice [28]. Therefore, training programs with a clear focus on enhancing specific clinical skills are generally more effective in improving clinical performance and patient outcomes.

The study demonstrated that PACE is acceptable. There is strong acceptance and understanding among providers that PACE should become part of their normal work. This cognitive participation reflects a high level of engagement and willingness to incorporate PACE into daily routines. Providers recognized the value of PACE in improving their knowledge and skills, with many noting that the program had significantly enhanced their understanding and management of seriously ill children. They also found PACE useful in training medical students and junior doctors, indicating its potential for broader educational impact. This widespread acceptance and integration into daily work routines suggest that PACE is viewed not just as an additional resource but as a vital component of their professional development.

When individuals perceive that a new activity should become part of their normal work, it is often associated with increased usage and integration into their daily routines [29, 30]. This concept, known as cognitive participation, reflects a high level of engagement and commitment, which positively influences the adoption and sustained use of new practices. For instance, a study by May et al. (2009) on Normalization Process Theory highlighted that when healthcare providers viewed new clinical practices as integral to their work, they were more likely to implement them consistently [31]. Similarly, if people recognize the value of a new activity, there is substantial evidence that this recognition leads to increased usage and behavior change. Michie et al. (2011) found that perceived usefulness and perceived ease of use are significant predictors of the intention to use and actual usage of new interventions [32]. Furthermore, Rogers’ Diffusion of Innovations theory (2003) emphasizes that when individuals see clear benefits and value in a new practice, they are more likely to adopt it, leading to a transformation in their behavior and routines [33]. These findings collectively suggest that cognitive acceptance and perceived value are critical drivers of the successful implementation and sustained usage of new activities in various contexts.

However, challenges to scalability remain, particularly in ensuring adequate resource and infrastructure support. While the program itself is well-received, practical barriers such as adequate time to complete adaptive e-learning or participate in skills practice sessions and health system internet support hinder its full implementation. In addition, providers reported difficulties in accessing necessary equipment and managing cases during power outages, which directly impact their ability to apply PACE training effectively and cement long-term knowledge and skills. These challenges highlight the need for systemic improvements in resource allocation and infrastructure to support the sustainable and effective integration of PACE into the healthcare system. Without addressing these critical barriers, the scalability of PACE may be limited, preventing it from reaching its full potential impact.

Three strategies would address these challenges: 1) Strengthening Digital Infrastructure, 2) Flexible Scheduling and Time Management, and 3) Provision of Essential Equipment and Resources.

#### Strengthening digital infrastructure

Investing in robust digital infrastructure is crucial for the successful implementation of e-learning programs. Ensuring reliable internet connectivity and access to digital devices can significantly enhance the feasibility of adaptive learning modules. UNICEF’s conducted a review of digital learning programs in low-resource settings that highlights the positive impact of improved digital infrastructure [34]. Additionally, providing technical support and maintenance can prevent disruptions and ensure the smooth operation of online learning platforms (Aranda-Jan et al., 2014).

#### Flexible scheduling and time management

Allowing healthcare providers flexible scheduling to complete adaptive e-learning modules and participate in skills practice sessions can mitigate time-related barriers. Research by Yardley et al. (2012) demonstrates that flexible learning schedules increase participation and completion rates in professional development programs. Implementing self-paced learning options and modular training formats can help healthcare providers integrate training into their busy schedules without compromising clinical duties.

#### Provision of essential equipment and resources

Ensuring the availability of necessary medical equipment and resources is essential for the practical application of training programs. Partnerships with governmental and non-governmental organizations can facilitate the procurement and distribution of essential tools. A study by Bertram et al. (2018) suggests that strategic resource allocation and collaborative efforts can address equipment shortages and improve healthcare delivery. Additionally, creating contingency plans for managing power outages, such as providing backup power solutions, can enhance the reliability of training programs in resource-limited settings.

Qualitative data from focus groups and interviews enriched the findings by providing detailed insights that both supported and contrasted with the NoMAD survey results. These qualitative insights highlighted the strengths of PACE, such as its alignment with Tanzanian guidelines and its educational value, while also revealing challenges like resource constraints. Providers shared specific examples of how PACE had positively impacted their clinical practice, such as improving their ability to manage emergencies and enhancing their confidence in providing care. However, they also pointed out the difficulties in sustaining PACE’s benefits without adequate support and clinical resources to translate this knowledge into improved care delivery. This mixed-methods approach offered a comprehensive understanding of the implementation process, emphasizing the importance of addressing both the strengths and weaknesses of PACE in a resource-limited setting. The contrast between the high satisfaction reported in surveys and the practical challenges discussed in interviews underscores the need for a mixed methods approach when implementing new complex interventions in such environments.

### Implications for implementation science and pediatric acute care

This study highlights the utility of the NPT as a conceptual framework for understanding the complexities involved in implementing adaptive learning interventions in LMICs. The findings provide valuable insights into the various factors that influence the implementation of adaptive learning, which can be applied to other healthcare interventions.

For pediatric acute care, the strong agreement among healthcare providers on the benefits of PACE for managing specific pediatric cases suggests that the program could significantly enhance provider proficiency and improve patient outcomes. Given the often time-sensitive nature of pediatric acute care, where timely and effective interventions such as oxygen therapy, intravenous fluids, and anti-microbial therapy can have a significant impact on patient outcomes, the effective and efficient training provided by PACE could lead to improved patient outcomes. Additionally, the consistency of these findings across various implementation contexts points to the scalability of the program, indicating its potential to be effectively expanded to other healthcare settings.

### Limitations

The study has several limitations. The small sample size limits the generalizability of the findings, and the low response rate of 30% may introduce response bias. Additionally, the study's short duration did not allow for a comprehensive assessment of all NPT constructs, particularly reflexive monitoring. The reliance on self-reported data may also introduce social desirability bias. Our mixed methods approach, and methodological triangulation enhance the robustness of the findings despite these limitations.

### Recommendations for future research

Future research should focus on longitudinal studies to assess the long-term sustainability and impact of PACE on provider proficiency, patient outcomes, and the quality of care. More rigorous qualitative research designs, such as detailed case studies and ethnographic studies, could provide a deeper understanding of the challenges and opportunities associated with implementing PACE. Additionally, research should explore the scalability of PACE, assessing how the program can be adapted for different healthcare settings and evaluating the resource implications of scaling up the intervention.

## Conclusions

This study offers valuable insights into the feasibility, acceptability, and scalability of implementing PACE in a Tanzanian context. While PACE aligns well with healthcare objectives, addressing resource and infrastructure challenges is crucial for its effective and sustainable implementation. The study underscores the value of the NPT as a framework for guiding implementation processes, with broader implications for implementation science and pediatric acute care in LMICs. Future researchers can apply these insights by ensuring alignment with facility goals, engaging stakeholders early, planning for long-term evaluations, addressing resource challenges proactively, and considering the specific context and available resources when assessing scalability.

## Supplementary Information

Supplementary Material 1.

## Data Availability

Deidentified participant data from this study are available upon reasonable request. Interested researchers may obtain the data by contacting the corresponding author, Dr. Peter Meaney, at meaneypa@stanford.edu. Access to the data will be granted following approval by an independent review committee established to evaluate the scientific validity and ethical justification of the proposed use of the tool. Please note that only the deidentified participant data are available, and no additional supporting information, such as study protocols or statistical analysis plans, will be provided. This process ensures that the data are used responsibly and in accordance with ethical research standards.

## Abbreviations

BMC: Bugando Medical Centre
e-learning: Electronic Learning
FGD: Focus Group Discussion
IDI: In-Depth Interview
LMIC: Low- or -Middle-Income Country
NPT: normalization process theory
NoMAD: normalization measure development
PACE: Pediatric Acute Care Education

## Data availability

Deidentified participant data from this study are available upon reasonable request. Interested researchers may obtain the data by contacting the corresponding author, Dr. Peter Meaney, at meaneypa@stanford.edu. Access to the data will be granted following approval by an independent review committee, established to evaluate the scientific validity and ethical justification of the proposed use. Please note that only the deidentified participant data is available, and no additional supporting information, such as study protocols or statistical analysis plans, will be provided. This process ensures that the data is used responsibly and in accordance with ethical research standards.
