# Supplementary material for: Evaluating the implementation of the Pediatric Acute Care Education (PACE) program in northwestern Tanzania: a mixed-methods study guided by normalization process theory
Source: BMC Health Serv Res. 2024 Sep 13;24:1066. doi: 10.1186/s12913-024-11554-3 (PMC11401409; doi:10.1186/s12913-024-11554-3)
Supplement: Supplementary file 1 — Supplementary Material 1. [file 12913_2024_11554_MOESM1_ESM.docx]

# Supplementary Materials

## NOMAD Survey Instrument

##

## Interview Guides

### Focus Group Interview Guide – English

| **PACE: Focus Group Discussion Guide for Junior Health Care Providers** |
| --- |
| **Background data on focus groups and providers**   \| PACE Focus Group Identification number: \| \| \| \| \| \| --- \| --- \| --- \| --- \| --- \| \| Type of Group (e.g., age, sex): \| \| \| \| \| \| Date: \| \| \| \| \| \| Location: \| \| \| \| \| \| Description of place where discussion took place: \| \| \| \| \| \| Facilitator name: \| \| \| \| \| \| Recorder’s/Notes taker’s name: \| \| \| \| \| \| Start / finish time: \| \| \| \| \| \| Providers \| \| \| \| \| \|  \| Provider’s ID number \| Sex \| Age \| Education \| Occupation \| Marital status \| \| 1 \|  \|  \|  \|  \|  \|  \| \| 2 \|  \|  \|  \|  \|  \|  \| \| 3 \|  \|  \|  \|  \|  \|  \| \| 4 \|  \|  \|  \|  \|  \|  \| \| 5 \|  \|  \|  \|  \|  \|  \| \| 6 \|  \|  \|  \|  \|  \|  \| \| 7 \|  \|  \|  \|  \|  \|  \| \| 8 \|  \|  \|  \|  \|  \|  \|   **Instructions to facilitator/moderator**  *After each open question* *there are a series of probes – please use these appropriately to tease out spontaneous discussion among providers– encouraging providers to talk among themselves-asking whichever are needed to get information that the providers have not already given in response to the open question – take special care not to miss critical probes like those related to “critical care”*  **Background**  1.What are your professional backgrounds and current roles?  Probe 1.1 Each provider has to briefly describe what one usually does on daily basis as part of job description.  2. Before PACE, what were your understanding of a seriously ill children?  Probe 2.1 Before PACE, What were your involvement in caring for seriously ill children?  Probe 2.2 Before PACE, How were the seriously ill children recognized and treated differently from other hospitalized children?  Probe 2:3 Before PACE, Are there any patients/groups you didn’t treat in this way?  Probe 2.4 Before PACE, What did you intend to achieve from treating seriously ill children?  **Evaluation of the use and implementation of PACE using the 4 NPT constructs**  ***Coherence***  3. How does PACE help you in your day to day job routine?  Probe 3.1What is the purpose of PACE?  Probe 3.2What is the benefit of PACE?  Probe 3.3 How will PACE fit within the overall goals and activities of the organization?  ***Cognitive Participation***  4. What are your professional opinion of pediatric acute care education (PACE) intervention?  Probe 4.1 How committed are you (in terms of time and energy) with PACE? (How many times per day/week)  Probe 4.2 How do you engage with PACE as an individual or with colleagues?  Probe 4.3 What are the best ways to study PACE?  Probe 4.4 What are the best ways of using PACE as an intervention?  ***Collective Action***   1. Explain how PACE was cascaded to other health care providers?   Probe 5.1What were the barriers to effective translation of PACE into practice?  Probe 5.2 What were the facilitators to effective translation of PACE into practice and compatibility  with pediatric acute care standard operating procedure?  Probe 5.3 How confident do you feel about PACE as a tool for equipping providers with the necessary knowledge and skills to deliver high quality care in comparison to conventional training?  Probe 5.4 Who do you feel ought to have the responsibility of overseeing the administration of PACE to learners?  Probe 5.5 What are the impact of the different types of feedback (email, WhatsApp, verbally, in person with coordinator, peers or your supervisor) on your participation in PACE over time?  Probe 5.6 What are the impact of PACE on resources, responsibility between health care providers and other health professionals?  ***Reflexive Monitoring***   1. How has PACE affected you in providing clinical services in your work environment?   Probe 6.1: Has your knowledge of care of seriously ill children changed as a result of using PACE? Please provide examples  Probe 6.2: Have your skills to provide care of seriously ill children changed as a result of using PACE? Please provide examples  Probe 6.3: Has PACE affected the outcomes of seriously ill children? Please provide examples  Probe 6.4: Has PACE affected how you interact with colleagues towards delivering care to seriously ill children? Please provide examples  Probe 6.5: Describe any alterations you or your colleagues have implemented to make it easier to use PACE  Probe 6.6: In what ways could the PACE be improved/adapted? Explain   1. Do you have anything else to share with us or any question regarding our discussion?   **We have come to an end of our discussion. Thank you for your time and insights.** |

### Focus Group Interview Guide – Swahili

| **PACE-Mwongozo wa majadiliano katika vikundi [Kwa watumishi wa afya- Juniors]** |
| --- |

**Taarifa za utangulizi za majadiliano na za washiriki**

| Namba ya utambulisho wa kikundi | | | | |  | | | |
| --- | --- | --- | --- | --- | --- | --- | --- | --- |
| Aina ya kikundi (Umri, Jinsi) | | | | |  | | | |
| Tarehe ya majadiliano | | | | |  | | | |
| Mahali | | | | |  | | | |
| Maelezo ya mahali majadiliano yalipofanyika | | | | |  | | | |
| Majina ya muwezeshaji | | | | |  | | | |
| Majina ya mtunza kumbukumbu | | | | |  | | | |
| Muda wa kuanza/Kumaliza majadiliano | | | | |  | | | |
| Taarifa za washiriki | | | | | | | | |
|  | Namba ya mshiriki | Jinsi | Umri | Kiwango cha juu cha elimu | Kazi/Shughuli | Hali ya ndoa | Madhehebu | Kabila |
| 1 |  |  |  |  |  |  |  |  |
| 2 |  |  |  |  |  |  |  |  |
| 3 |  |  |  |  |  |  |  |  |
| 4 |  |  |  |  |  |  |  |  |
| 5 |  |  |  |  |  |  |  |  |
| 6 |  |  |  |  |  |  |  |  |
| 7 |  |  |  |  |  |  |  |  |
| 8 |  |  |  |  |  |  |  |  |

**Maelekezo kwa Wawezeshaji.**

***Baada ya kila swali kuna maswali ya kudadisi*** *– tafadhali yatumie maswali hayo ipasavyo – yaulize yanayohitajika kupata taarifa ambazo mhojiwa hajazitoa kwenye maswali ya msingi – chukua tahadhari maalum ili usikose kudadisi taarifa zinazohusu “critical care”*

**Utangulizi.**

1.Je ninyi mna taaluma gani na mnafanya kazi kama akina nani (majukumu) yenu kwa sasa ni yapi?

*Dadisi* *1.1* *Elezeni kwa kifupi majukumu yenu ya kila siku ni yapi kama sehemu ya maelezo ya kazi zenu*

2. *Kabla ya mafunzo ya PACE, Je mlikuwa mnaelewa nini kuhusu mtoto mgonjwa anaehitaji* *uangalizi wa karibu /haraka?*

*Dadisi* 2.1 Kabla ya mafunzo ya PACE, Je mlikuwa mnahusikaje katika kutoa huduma kwa watoto wanaohitaji uangalizi wa karibu/haraka (seriously ill children)?

*Dadisi 2.2* Kabla ya mafunzo ya PACE, *Je watoto wagonjwa wanaohitaji uangalizi wa karibu*(seriously ill children) *walitambuliwaje na kutibiwa tofauti na waliolazwa?*

*Dadisi 2.3* Kabla ya mafunzo ya PACE, Je kuna watoto wagonjwa wowote ambao hamkuwatibu *kwa namna hii? (Rejea majibu ya 2.2)*

*Dadisi 2.4* Kabla ya mafunzo ya PACE*,Je mlikuwa mnakusudia nini kwa kuwatibu watoto wagonjwa wanaohitaji uangalizi wa karibu/haraka (seriously ill children)?*

Tathmini ya matumizi na utekelezaji wa mafunzo ya PACE kwa kutumia 4NPT constructs:

***Coherence***

3. Je mafunzo ya PACE yanawasaidiaje katika utendaji wenu wa kazi wa kila siku?

*Dadisi 3.1 Je madhumuni ya mafunzo ya PACE ni yapi?*

*Dadisi 3.2 Je mafunzo ya PACE yana manufaa gani?*

*Dadisi 3.3 Je mafunzo ya PACE yanaendana vipi(yanafananaje) na malengo ya ujumla na shughuli za taasisi yenu?*

***Kushiriki katika Utambuzi***

4. Je mna maoni gani ya kitaalam kuhusu afua (intervention) ya PACE?

*Dadisi 4.1 Je ni kwa namna gani mmejitoa (kutenga muda*-mara ngapi kwa siku/wiki *na nguvu) kwa ajili ya mafunzo ya PACE?*

*Dadisi 4.2 Je mnajihusisha vipi na mafunzo ya PACE mkiwa mmoja mmoja au mkiwa na wenzenu?*

*Dadisi 4.3 Je ni njia ipi bora zaidi ya kusoma PACE?*

*Dadisi 4.4 Je ni njia bora zaidi ya kutekeleza PACE?*

***Hatua jumuishi***

5. Elezeni ni kwa namna gani mafunzo ya PACE yalivyowafikia watumishi wa afya?

*Dadisi 5.1 Je kulikuwa na vikwazo gani katika kutafsiri mafunzo ya PACE kwa vitendo?*

*Dadisi 5.2 Je mambo gani yaliwawezesha kuyapeleka mafunzo ya PACE kwenye vitendo na kukidhi taratibu za kiutendaji (SOP) za watoto wagonjwa wanaohitaji uangalizi wa karibu na haraka?*

*Dadisi 5.3 Je mnahisi kujiamini vipi kuhusu mafunzo ya PACE kama zana ya kuwapa watumishi maarifa na ujuzi ili kutoa huduma bora zaidi mkilinganisha na mafunzo ya kawaida?*

*Dadisi 5.4 Je mnafikiri wakina nani wanapaswa kuwa na majukumu ya kusimamia mafunzo ya PACE kwa wanaotaka kujifunza(watumiaji)?*

*Dadisi 5.5 Je matumizi ya aina tofauti za mrejesho (barua pepe, Whatsapp, wa maneno, na kukutana ana kwa ana na waratibu, rika au wasimamizi wenu) yamekuwa na matokeo gani katika ushiriki wenu kwenye mafunzo ya PACE?*

*Dadisi 5.6 Je mafunzo ya PACE yamekuwa na matokeo gani kuhusu mgawanyo wa raslimali na wajibu kati ya watumishi wa afya na wataalam wengine wa afya?*

***Ufuatiliaji wa kujitathmini***

6. Je mafunzo ya PACE yamekuwa na matokeo gani katika utoaji wenu wa huduma?

*Dadisi 6.1 Je maarifa yenu ya uangalizi wa karibu na haraka kwa watoto wagonjwa yamebadilika kutokana na kutumia mafunzo ya PACE? Tafadhali toeni mifano*

*Dadisi 6.2 Je ujuzi wenu wa kutoa uangalizi kwa watoto wagonjwa wanaohitaji uangalizi wa karibu na haraka umebadilika kutokana na kutumia mafunzo ya PACE? Tafadhali toeni mifano*

*Dadisi 6.3 Je mafunzo ya PACE yamebadili matokeo ya hali za watoto wagonjwa* *wanaohitaji uangalizi wa karibu na haraka? Tafadhali toeni mifano*

*Dadisi 6.4 Je mafunzo ya PACE yamebadili namna mnavyo changamana na wenzenu katika kutoa uangalizi wa watoto wagonjwa wanaohitaji uangalizi wa karibu na haraka? Tafadhali toa mifano*

*Dadisi 6.5 Elezeni kuhusu mabadiliko yoyote ambayo ninyi au wenzenu mmeyatekeleza/wameyatekeleza kurahisisha matumizi ya mafunzo ya PACE?*

*Dadisi 6.6 Je ni kwa namna gani mafunzo ya PACE yanaweza kuboreshwa/kutumika?*

7. Je mna jambo jingine ambalo mnataka kutuambia au mna swali lolote kuhusu majadiliano yetu?

**Tumefika mwisho wa majadiliano yetu. Ahsante kwa muda wenu na mawazo yenu mazuri.**

### Interview Guide – English

**PACE: Interview Guide for Health Care Providers (Seniors)**

**Background data on interview and informants**

| **The Location:** | |
| --- | --- |
| Name of health facility: |  |
| Level of the facility |  |
| Type of facility (e.g., hospital/health centre/dispensary): |  |
| Type of organization (e.g., government/private/NGO/church/other): |  |
| Actual location of interview (e.g., Health Facility/Non Health Facility etc.): |  |
| Date of interview: |  |
| Time of interview: Start / Finish and Duration |  |
| **The informant:** | |
| Interviewee’s ID No. |  |
| Sex (male/female): |  |
| Highest level of education (e.g., Certificate, diploma, degree, post grad degree): |  |
| Designation (e.g., Medical Officer, Clinical officer, Nurse, etc.): |  |
| Current medical specialization – if any: |  |
| Past experience/specialisms: |  |
| Years of work at present health facility: |  |
| Years of medical practice in total: |  |
| **The interviewer:** | |
| Name(s) of interviewer(s): |  |

1.What is your professional background and current role?

2. Before PACE, what was your understanding of a seriously ill child?

Probe 2.1 Before PACE, what is your involvement in caring for seriously ill children?

Probe 2.2 Before PACE, how are the seriously ill children recognized and treated differently from other hospitalized children?

Probe 2.3 Before PACE, were there any seriously sick children that you didn’t treat this way?

Probe 2.4 Before PACE, what do you intend to achieve from treating seriously ill children?

**Evaluation of the use and implementation of PACE using the 4 NPT constructs**

***Coherence***

3. How does PACE help you in your day to day job routine?

Probe 3.1What is the purpose of PACE?

Probe 3.2 What is the benefit of PACE?

Probe 3.3 How will PACE fit within the overall goals and activities of the organization?

***Cognitive Participation***

4. What is your professional opinion of pediatric acute care education (PACE) intervention?

Probe 4.1 How committed are you (in terms of time and energy) with PACE? (How many times per day/week)

Probe 4.2 How do you engage with PACE as an individual or with colleagues?

Probe 4.3 What is the best way to study PACE?

Probe 4.4 What is the best way of using PACE as an intervention?

***Collective Action***

1. Explain how PACE was cascaded to other health care providers?

Probe 5.1 What were the barriers to effective translation of PACE into practice?

Probe 5.2 What were the facilitators to effective translation of PACE into practice and compatibility with pediatric acute care standard operating procedure?

Probe 5.3 How confident do you feel about PACE as a tool for equipping providers with the necessary knowledge and skills to deliver high quality care in comparison to conventional training?

Probe 5.4 Who do you feel ought to have the responsibility of overseeing the administration of PACE to learners?

Probe 5.5 What was the impact of the different types of feedback (email, whatsapp, verbally, in person with coordinator, peers or your supervisor) on providers participation in PACE over time?

Probe 5.6 What was the impact of PACE on the allocation of resources and responsibility between health care providers and other health professionals?

***Reflexive Monitoring***

1. How has PACE affected you in providing clinical services in your work environment?

Probe 6.1: Has your knowledge of care of seriously ill children changed as a result of using PACE? Please provide examples

Probe 6.2: Have your skills to provide care of seriously ill children changed as a result of using PACE? Please provide examples

Probe 6.3: Has PACE affected the outcomes of seriously ill children? Please provide examples

Probe 6.4: Has PACE affected how you interact with colleagues towards delivering care to seriously ill children? Please provide examples

Probe 6.5: Describe any alterations you or your colleagues have implemented to make it easier to use PACE

Probe 6.6: In what ways could the PACE be improved/adapted? Explain

1. Do you have anything else to share with us or any question regarding our interview?

**We have come to an end of our interview. Thank you for your time and insights.**

### Interview Guide – Swahili

**PACE: Mwongozo wa mahojiano na watumishi wa afya (Waandamizi)**

**Taarifa za Utangulizi za Hojaji na za wahojiwa.**

| **Mahali:** | |
| --- | --- |
| Jina la kituo cha afya : |  |
| Ngazi (level) ya kituo cha afya: |  |
| Aina ya kituo cha afya (km., hospitali/ /Zahanati): |  |
| Aina ya Taasisi (k.m., Serikali/Binafsi/Shirika lisilo la Kiserikali/Kanisa/Nyingine): |  |
| Mahali pa Mahojiano(km. Health facility,Kwingine n.k.,): |  |
| Tarehe ya mahojiano: |  |
| Muda wa mahojiano: Kuanza / Kumaliza Muda uliotumika kufanya mahojiano |  |
| **Taarifa za mhojiwa:** | |
| Namba ya utambulisho ya mhojiwa |  |
| Jinsi (mwanaume/mwanamke): |  |
| Kiwango cha juu cha elimu (k.m., Astashahada, Stashahada, Shahada, Shahada ya uzamili): |  |
| Cheo (k.m.,Mganga msaidizi, Mganga, Tabibu, Muuguzi, n.k.,): |  |
| Ubobezi– kama amebobea: |  |
| Uzoefu wa siku za nyuma/Utaalam: |  |
| Muda uliodumu katika kituo cha kazi cha sasa: |  |
| Muda uliodumu katika kazi kwa ujumla: |  |
| **Mhojaji** | |
| Jina/Majiana ya Mhojaji: |  |

**Maelekezo kwa wahojaji**

***Baada ya kila swali kuna maswali ya kudadisi*** *– tafadhali yatumie maswali hayo ipasavyo – yaulize yanayohitajika kupata taarifa ambazo mhojiwa hajazitoa kwenye maswali ya msingi – chukua tahadhari maalum ili usikose kudadisi taarifa zinazohusu “critical care”*

**Utangulizi.**

1.Je wewe una taaluma gani na unafanya kazi kama nani (jukumu) lako kwa sasa ni lipi?

***Dadisi*** *1.1* *Eleza kwa kifupi majukumu yako ya kila siku ni yapi kama sehemu ya maelezo ya kazi yako*

2. *Je kabla ya mafunzo ya PACE, uelewa wako wa mtoto mgonjwa mahututi ni upi*

*Dadisi* 2.1 Je ulikuwa unahusikaje katika kutoa huduma kwa watoto wanaohitaji uangalizi wa karibu (seriously ill children) (kabla ya mafunzo ya PACE)?

*Dadisi 2.2* Kabla ya mafunzo ya PACE, *Je watoto wagonjwa wanaohitaji uangalizi wa karibu*(seriously ill children) *wanatambuliwaje na kutibiwa tofauti na waliolazwa?*

*Dadisi 2.3* Kabla ya mafunzo ya PACE, Je kuna mtoto mgonjwa yoyote ambae haukumtibu *kwa namna hii? (Rejea majibu ya 2.2)*

*Dadisi 2.4* Kabla ya mafunzo ya PACE*,Je ulikusudia nini kwa kuwatibu watoto wagonjwa wanaohitaji uangalizi wa karibu/haraka (seriously ill children)*

Tathmini ya matumizi na utekelezaji wa mafunzo ya PACE kwa kutumia 4NPT constructs:

***Coherence***

3. Je mafunzo ya PACE yanakusaidiaje katika utendaji wako wa kazi wa kila siku?

*Dadisi 3.1 Je madhumuni ya mafunzo ya PACE ni yapi?*

*Dadisi 3.2 Je mafunzo ya PACE yana manufaa gani?*

*Dadisi 3.3 Je mafunzo ya PACE yanaendana vipi(yanafananaje) na malengo ya ujumla na shughuli za taasisi yako?*

***Kushiriki katika Utambuzi***

4. Je nini maoni yako ya kitaalam kuhusu afua ya PACE?

*Dadisi 4.1 Je umejitoa (kutenga muda-* mara ngapi kwa siku/wiki *na nguvu) kwa ajili ya mafunzo ya PACE?*

*Dadisi 4.2 Je unajihusisha vipi na mafunzo ya PACE wewe binafsi na wenzako?*

*Dadisi 4.3 Je ni njia ipi bora zaidi ya kusoma PACE?*

*Dadisi 4.4 Je ni njia ipi bora zaidi ya kutekeleza PACE?*

***Hatua jumuishi***

5. Eleza ni kwa namna gani mafunzo ya PACE yalivyowafikia watumishi wa afya?

*Dadisi 5.1 Je kulikuwa na vikwazo gani katika kutafsiri mafunzo ya PACE kwa vitendo*

*Dadisi 5.2 Je mambo gani yaliwezesha kuyapeleka mafunzo ya PACE kwenye vitendo na kukidhi taratibu za kiutendaji (SOP) za watoto wagonjwa wanaohitaji uangalizi wa karibu na haraka?*

*Dadisi 5.3 Je unahisi kujiamini vipi kuhusu mafunzo ya PACE kama zana ya kuwapa watumishi maarifa na ujuzi ili kutoa huduma bora zaidi ukilinganisha na mafunzo ya kawaida?*

*Dadisi 5.4 Je unafikiri ni nani wanapaswa kuwa na majukumu ya kusimamia mafunzo ya PACE kwa anaetaka kujifunza(watumiaji)?*

*Dadisi 5.5 Je matumizi ya aina tofauti za mrejesho (barua pepe, Whatsapp, wa maneno, na kukutana ana kwa ana na waratibu, rika au msimamizi wako) yamekuwa na matokeo gani katika ushiriki wako kwenye mafunzo ya PACE?*

*Dadisi 5.6 Je mafunzo ya PACE yamekuwa na matokeo gani kuhusu mgawanyo wa raslimali na wajibu kati ya watumishi wa afya na wataalam wengine wa afya ?*

***Ufuatiliaji wa kujitathmini***

6. Je mafunzo ya PACE yamekuwa na matokeo gani katika utoaji wako wa huduma?

*Dadisi 6.1 Je maarifa yako ya uangalizi wa karibu na haraka kwa watoto wagonjwa yamebadilika kutokana na kutumia mafunzo ya PACE? Tafadhali toa mifano*

*Dadisi 6.2 Je ujuzi wako wa kutoa uangalizi kwa watoto wagonjwa wanaohitaji uangalizi wa karibu na haraka umebadilika kutokana na kutumia mafunzo ya PACE? Tafadhali toa mifano*

*Dadisi 6.3 Je mafunzo ya PACE yamebadili matokeo ya hali za watoto wagonjwa wanaohitaji uangalizi kwa karibu na haraka? Tafadhali toa mifano*

*Dadisi 6.4 Je mafunzo ya PACE yamebadili namna unavyo changamana na wenzako katika kutoa uangalizi wa watoto wagonjwa mahututi? Tafadhali toa mifano*

*Dadisi 6.5 Eleza kuhusu mabadiliko yoyote ambayo wewe au wenzako umeyatekeleza/wameyatekeleza kurahisisha matumizi ya mafunzo ya PACE*

*Dadisi 6.6 Je ni kwa namna gani mafunzo ya PACE yanaweza kuboreshwa/kutumika?*

7. Je una jambo jingine ambalo unataka kutuambia au una swali lolote kuhusu mahojiano yetu?

**Tumefika mwisho wa mahojiano yetu. Ahsante kwa muda wako na mawazo yako mazuri.**

## Focus Group Protocols

*Interview guides development description.* Development of interview guides was based on face-to-face in-depth interviews (IDIs) and focus group discussions (FGDs) designed to capture healthcare providers’ experiences and interpretations on their own terms while ensuring the key themes from the IDI and FGD guides were fully explored. Prior to data collection, research design and tools were developed collectively by study investigators. Training and pretesting of tools were offered to six research assistants who were involved in data collection (3 males and 3 females) with medical and social science backgrounds by a social scientist and a paediatrician from CUHAS from 19^th^ to 23^rd^ September 2022.

*Data collection process.* IDIs and FGDs were conducted using guides in Kiswahili. The interviews lasted between 30 to 45 minutes whereas FGDs between 1hour and thirty minutes to 2 hours and were conducted at the premises where the providers worked. The content of interviews and focus groups were the same for the sake of methodological triangulation.(33) The guides included questions about socio-demographic characteristics, background information, evaluation of the use and implementation of PACE using the NPT constructs: coherence, cognitive participation, collective action, and reflexive monitoring.

Conduction of interviews and focus groups at providers’ work premises allowed us to contextualize data. Towards the end of interviews and discussions, providers were invited to provide additional information and were given opportunity to ask questions, if any.

*Conduct and recording of discussions and interviews:* Conduction of interviews and focus groups at providers’ work premises allowed us to contextualize data. Towards the end of interviews and discussions, providers were invited to provide additional information and were given opportunity to ask questions, if any.

Interviews and discussions were digitally recorded (with the permission from the providers) and hand-written notes were also taken to complement the recordings. Fieldnotes were written up in a form of fully-fledged field notes immediately after each interview or discussion and provided a detailed description of the interview/discussion situation, including additional information which could not be recorded, such as non-verbal cues and other interactions.

*Qualitative analysis techniques for focus group discussions and interviews.* Management and analysis of qualitative data was an iterative process.(26) All 24 and 13 audio files containing interviews and discussions respectively were transferred into the computer from digital recorders, backed-up in another computer in the office, and transcribed verbatim. *Kiswahili* transcripts were translated in English for further analysis. The English transcripts were subsequently checked against the audios for accuracy of the translation. Back translation was done by randomly selecting a few English transcripts and then translating them back to *Kiswahili*. Hand-written notes taken during the interviews and discussions were subsequently typed up and included in the analysis. Transcripts were coded by two investigators to minimize inter-coder variability using NVivo 2020 qualitative data analysis (QSR International Pty Ltd. Sydney, Australia) software.

Data analysis was carried out stage-wise, using a combination of deductive and inductive coding (also called “hybrid” coding).(34) The *code system* (and the categories and the themes that were developed based on coding process) was developed gradually and collaboratively. Data was organized in a systematic and structured way by using topics in the interview guide to initially categorize and code the data (deductive coding); those topics became the initial codes. The data was systematically and iteratively reviewed by looking at specific information related to topics to ensure an exhaustive set of data support each code.

Data was also coded inductively as suggested by Hennink and colleagues,(35,36) to allow coded to derive from the data and during this process, additional themes that were not captured by the interview guide were identified, and the internal validity and robustness of existing codes was confirmed. Data was then reduced into manageable sizes by identifying common themes, patterns, or categories. This involved grouping coded data into larger categories or themes.

Thematic content analysis was then conducted by two independent investigators. Data was then displayed in a way that is meaningful and accessible to others (in this case through text and quotations) that illustrates key themes. Conclusions from the data were also drawn by two investigators (to cross-check and validate interpretation of the data). Contextual information collected during the interviews and discussions helped us to interpret the emerging findings. Since processing and analysis of qualitative data was systematic, explicit, and reproducible, the validation and trustworthiness of the findings was established.(26)

In reporting this study, we have followed a checklist for explicit and comprehensive reporting of qualitative studies (in-depth interviews and focus groups).(27) Quotations throughout this report are illustrative of experiences reported by the interviewees.
